# Phylogenomic Classification and the Evolution of Clonal Complex 5 Methicillin-Resistant *Staphylococcus aureus* in the Western Hemisphere

**DOI:** 10.3389/fmicb.2018.01901

**Published:** 2018-08-22

**Authors:** Lavanya Challagundla, Jinnethe Reyes, Iftekhar Rafiqullah, Daniel O. Sordelli, Gabriela Echaniz-Aviles, Maria E. Velazquez-Meza, Santiago Castillo-Ramírez, Nahuel Fittipaldi, Michael Feldgarden, Sinéad B. Chapman, Michael S. Calderwood, Lina P. Carvajal, Sandra Rincon, Blake Hanson, Paul J. Planet, Cesar A. Arias, Lorena Diaz, D. Ashley Robinson

**Affiliations:** ^1^Department of Data Science, University of Mississippi Medical Center, Jackson, MS, United States; ^2^Molecular Genetics and Antimicrobial Resistance Unit, International Center for Microbial Genomics, Universidad El Bosque, Bogota, Colombia; ^3^Department of Microbiology and Immunology, University of Mississippi Medical Center, Jackson, MS, United States; ^4^Instituto de Investigaciones en Microbiología y Parasitología Médica, Universidad de Buenos Aires and Consejo Nacional de Investigaciones Ciencias y Tecnicas, Buenos Aires, Argentina; ^5^Department of Vaccine Evaluation, Instituto Nacional de Salud Pública, Cuernavaca, Mexico; ^6^Programa de Genómica Evolutiva, Centro de Ciencias Génomicas, Universidad Nacional Autónoma de México, Cuernavaca, Mexico; ^7^Public Health Ontario Laboratory, Toronto, ON, Canada; ^8^Department of Laboratory Medicine and Pathobiology, University of Toronto, Toronto, ON, Canada; ^9^Department of Cell and Systems Biology, University of Toronto, Toronto, ON, Canada; ^10^National Center for Biotechnology Information, National Institutes of Health, Bethesda, MD, United States; ^11^Broad Institute of MIT and Harvard, Cambridge, MA, United States; ^12^Section of Infectious Disease and International Health, Dartmouth–Hitchcock Medical Center, Lebanon, NH, United States; ^13^Division of Infectious Diseases and Center for Antimicrobial Resistance and Microbial Genomics, University of Texas Health Science Center, McGovern Medical School, Houston, TX, United States; ^14^Center for Infectious Diseases, School of Public Health, University of Texas Health Science Center, Houston, TX, United States; ^15^Children’s Hospital of Philadelphia, University of Pennsylvania, Philadelphia, PA, United States

**Keywords:** methicillin-resistant *Staphylococcus aureus*, MRSA, phylogenomics, convergent evolution, local adaptation

## Abstract

Clonal complex 5 methicillin-resistant *Staphylococcus aureus* (CC5-MRSA) includes multiple prevalent clones that cause hospital-associated infections in the Western Hemisphere. Here, we present a phylogenomic study of these MRSA to reveal their phylogeny, spatial and temporal population structure, and the evolution of selected traits. We studied 598 genome sequences, including 409 newly generated sequences, from 11 countries in Central, North, and South America, and references from Asia and Europe. An early-branching CC5-Basal clade is well-dispersed geographically, is methicillin-susceptible and MRSA predominantly of ST5-IV such as the USA800 clone, and includes separate subclades for avian and porcine strains. In the early 1970s and early 1960s, respectively, two clades appeared that subsequently underwent major expansions in the Western Hemisphere: a CC5-I clade in South America and a CC5-II clade largely in Central and North America. The CC5-I clade includes the ST5-I Chilean/Cordobes clone, and the ST228-I South German clone as an early offshoot, but is distinct from other ST5-I clones from Europe that nest within CC5-Basal. The CC5-II clade includes divergent strains of the ST5-II USA100 clone, various other clones, and most known vancomycin-resistant strains of *S. aureus*, but is distinct from ST5-II strain N315 from Japan that nests within CC5-Basal. The recombination rate of CC5 was much lower than has been reported for other *S. aureus* genetic backgrounds, which indicates that recurrence of vancomycin resistance in CC5 is not likely due to an enhanced promiscuity. An increased number of antibiotic resistances and decreased number of toxins with distance from the CC5 tree root were observed. Of note, the expansions of the CC5-I and CC5-II clades in the Western Hemisphere were preceded by convergent gains of resistance to fluoroquinolone, macrolide, and lincosamide antibiotics, and convergent losses of the staphylococcal enterotoxin p (*sep*) gene from the immune evasion gene cluster of phage ϕSa3. Unique losses of surface proteins were also noted for these two clades. In summary, our study has determined the relationships of different clades and clones of CC5 and has revealed genomic changes for increased antibiotic resistance and decreased virulence associated with the expansions of these MRSA in the Western Hemisphere.

## Introduction

Methicillin-resistant *Staphylococcus aureus* (MRSA) is among the leading causes of antibiotic-resistant bacterial infections in hospital and community settings (Centers for Disease Control and Prevention, 2013; World Health Organization, 2014). These infections can range in severity from superficial skin infections to life-threatening invasive infections such as sepsis, infective endocarditis, and osteomyelitis (Boucher et al., 2010). Different MRSA clones predominate in different geographic regions and can differ in the rates and types of infections that they cause (Grundmann et al., 2010; David et al., 2014). Over the past 20 years, MRSA related to multilocus sequence type (ST) 5, which is classified into clonal complex (CC) 5, have been among the most prevalent clones causing hospital-associated infections in the Western Hemisphere (Aires De Sousa et al., 2001; Christianson et al., 2007; Rodriguez-Noriega et al., 2010; Diekema et al., 2014; Arias et al., 2017; Tickler et al., 2017). CC5-MRSA clones can differ in their Staphylococcal Chromosomal Cassette *mec* (SCC*mec*) genetic element, which carries the resistance determinant to anti-staphylococcal β-lactams, and in gene content (Christianson et al., 2007; Monecke et al., 2011). The identification of MRSA clones based on ST-SCC*mec* types (Robinson and Enright, 2004) has allowed for more precise transnational communication and tracking of these clones, and characterization of gene content has provided additional markers and insights into the lifestyles of these clones (Monecke et al., 2011).

Studies have identified the ST5-I Chilean/Cordobes clone (Sola et al., 2002), the ST5-II Canadian MRSA-2, New York/Japan, and USA100 clones (Roberts et al., 1998; McDougal et al., 2003; Christianson et al., 2007), and the ST5-IV USA800 clone (McDougal et al., 2003), as particularly common in the Western Hemisphere. For example, in the United States, ST5-II may have accounted for >40% of MRSA infections since the mid-1990s (Roberts et al., 1998; McDougal et al., 2003). In the southern cone of South America, ST5-I may have accounted for >60% of MRSA infections since the early 2000s (Sola et al., 2006). More recently in this region of South America, ST5-IV with *pvl* and *sea* toxin genes have emerged as a cause of community-associated infections and hospital infections especially among children (Sola et al., 2008; Egea et al., 2014). Other CC5-MRSA clones with SCC*mec* types I–VII have been reported elsewhere in the world (Monecke et al., 2011). In some cases, such as the ST225-II Rhine-Hesse clone in Central Europe (Nubel et al., 2010) and ST5-II in Western Australia (Coombs et al., 2007), the Western Hemisphere has been implicated as the origin of the clones.

Importantly, CC5 is the principal genetic background within *S. aureus* upon which full resistance to vancomycin—one of the drugs of choice for treating MRSA infections—repeatedly has arisen by acquisition of the *vanA* operon from *Enterococcus* spp. (Kos et al., 2012). The reasons why CC5 and not other *S. aureus* backgrounds has been the focal point for these acquisitions are not clear, but could include increased promiscuity, a genome that is conducive to *vanA* expression, or a shared niche with *Enterococcus* spp. In addition to the substantial disease caused by CC5-MRSA in human populations, the CC5 background has adapted to bird species and become a source of infection among broiler poultry (Lowder et al., 2009). The CC5 background also has been isolated from pigs (Frana et al., 2013), and with some traits that are present in other pig-adapted MRSA backgrounds (Hau et al., 2015).

The phylogenetic relationships between the numerous CC5-MRSA clones have not been determined, nor have the evolutionary events that led to their emergence. CC8 is the rival of CC5 for prominence among MRSA in the Western Hemisphere, and CC8-MRSA clone relationships and evolution have been studied extensively through genome sequencing (Uhlemann et al., 2014; Alam et al., 2015; Planet et al., 2015; Glaser et al., 2016; Challagundla et al., 2018). Relatively small samples of CC5-MRSA have been similarly studied (Kos et al., 2012; Aanensen et al., 2016; Arias et al., 2017). Here, we provide the first phylogenomic study of CC5-MRSA that is focused on large samples from the Western Hemisphere. Our goals are to resolve the phylogeny, place and time of origin of major CC5-MRSA clones, and to trace the evolution of their key traits.

## Materials and Methods

### Bacterial Samples and Genome Sequencing

The genome sequences of 598 strains of CC5 were studied here. Sequences were newly generated for 409 strains, and published sequences were downloaded from publicly available sources for 189 strains. The newly generated sequences were from three sequencing facilities: the Broad Institute (*n* = 297), the University of Mississippi Medical Center (UMMC, *n* = 74), and Universidad El Bosque (UEB, *n* = 38). Information on these sequences is provided in **Supplementary Table S1**. All new sequences were generated using Illumina instruments. The Broad Institute’s sequencing procedures are described in **Supplementary Text S1**, whereas previously published procedures were used at UMMC (Challagundla et al., 2018) and UEB (Arias et al., 2017). Eleven countries in the Western Hemisphere were sampled: Argentina, *n* = 30; Brazil, *n* = 23; Canada, *n* = 36; Chile, *n* = 23; Colombia, *n* = 56; Ecuador, *n* = 9; Guatemala, *n* = 21; Mexico, *n* = 40; Peru, *n* = 28; United States, *n* = 270; and Venezuela, *n* = 21. The US samples consisted of separate large collections: California, *n* = 40; Massachusetts, *n* = 40; Mississippi, *n* = 89; New York, *n* = 74; plus reference strains of USA100 and USA800, vancomycin-resistant *S. aureus* (VRSA), and avian and porcine strains. Other strains included completely sequenced reference strains JH1, N315, ED98, CF-Marseille, and 32 strains from Europe. Dates of isolation ranged from 1964 to 2017. Most strains were from clinical specimens.

### Genome Assembly, Pseudoread Generation, and Alignment

Some of the published sequences were available only as assembled genomes. For those published sequences available as reads, and for the new sequences generated by UMMC, assemblies were made after filtering reads for minimum quality (base quality ≥ Q13, number of ambiguities ≤ 2, read length ≥ 15) using CLC Genomics Workbench v7 (Qiagen, Aarhus, Denmark). For those new sequences generated by the Broad Institute, assembly was carried out using the ALLPATHS-LG pipeline^1^, followed by automated assembly improvement using Pilon^2^. Quality measures of all assemblies are listed in **Supplementary Table S1**.

In order to align this heterogeneous sample of sequences, we first used wgsim v0.3.2 (Li et al., 2009) with each assembly to generate 500,000 paired-end pseudoreads of 100 bp, with a 200 bp distance between the ends. These pseudoreads were mapped to the complete reference sequence of CC5 strain JH1 (Mwangi et al., 2007) using bwa v0.7.12 (Li and Durbin, 2010), coordinate-sorted with Picard v1.141 (DePristo et al., 2011), and realigned around indels with GATK v2.8-1 (DePristo et al., 2011).

### Variant Calling and Functional Effects

Single-nucleotide polymorphisms (SNPs) and short insertion–deletion polymorphisms (indels) were called with the UnifiedGenotyper walker of GATK. All self-similar sequences identified by pairwise megablast of the CC5 reference sequence against itself, plus five regions of mobile genetic elements, were excluded from variant calling. Mapping quality was >50 for all variants. The functional effects of the variants were determined using SnpEff v4 (Cingolani et al., 2012) with the annotation of the CC5 reference sequence.

### Strain Typing and Prediction of Antibiotic Resistance

Multilocus sequence typing (MLST) was done by scanning the strains’ genome assemblies against the *S. aureus* MLST database^3^, using the mlst v2.10 program^4^ with default settings. Typing of the SCC*mec* element was done by mapping the strains’ pseudoreads to a custom-clustered database of *ccr* and *mec* gene complexes, plus SCC*mec* IV subtype-specific sequences (Kaya et al., 2018), using the SRST2 v0.2.0 program (Inouye et al., 2014) with the min_coverage 60 option. The validity of this approach was investigated using pseudoreads generated from reference sequences of SCC*mec*^5^ and by manually checking non-typeable elements with the SCC*mec*Finder web-based tool (Kaya et al., 2018). The detection of antibiotic resistance genes and mutations and the corresponding predicted antibiotic resistance was done with Mykrobe predictor v0.1.3, which has shown high (>99%) sensitivity and specificity for predicting resistance of *S. aureus* to a panel of 12 antibiotics (Bradley et al., 2015).

### Detection of Selected Virulence Factors and Mobile Genetic Elements

To detect virulence factors, the full nucleotide dataset for *S. aureus* available at the Virulence Factors Database (Chen et al., 2016) was downloaded and then clustered into 265 groups with CD-HIT, using the tools available with SRST2 with default settings. The strains’ pseudoreads were mapped to this clustered database using SRST2 with the min_coverage 60 option. A similar approach was used to detect mobile genetic elements, specifically phages and integrative conjugative elements (ICEs) that integrate into the *S. aureus* chromosome. Phages were detected based on their integrase genes, using the 12 integrase groups described by Goerke et al. (2009) plus the integrase for ϕSPβ (Holden et al., 2010). ICEs were detected based on full-length sequences, using the seven subfamilies of ICE*6013* and two subfamilies of Tn*916* (Tn*916* and Tn*5801*) described by Sansevere and Robinson (2017) and Sansevere et al. (2017). The validity of this approach was investigated using pseudoreads generated from complete sequences of reference strains of CC1, CC5, CC8, CC30, CC151, and reference sequences of the phages and ICEs.

### Phylogenetic Inference and Recombination Detection

A maximum-likelihood (ML) phylogeny was constructed using PhyML v3.0 (Guindon et al., 2009) with the HKY+G model and five random and one BioNJ starting trees. For this analysis, an alignment representing biallelic SNPs and invariant core sequence present in all 598 genomes was used. Branch support was estimated with SH-like tests. The root of the tree was determined by first mapping reads from CC1, CC8, and ST72 outgroup sequences to the CC5 reference sequence, and then extracting the positions of the CC5 biallelic SNPs from the outgroups. These SNPs were then analyzed with distance-based (BioNJ) and maximum parsimony (MP) phylogenetic analysis. The outgroups consistently bisected a branch leading to a strain from Argentina, which was designated as the root. Recombination was detected using the PhyML tree with the method implemented in ClonalFrameML (CFML; Didelot and Wilson, 2015). Clade-specific recombination rates were examined on the recombination-corrected ML tree using R scripts.

### Molecular Clock Analysis

The R package of Murray et al. (2016) was used to verify that no confounding existed between the path lengths on the recombination-corrected ML tree and the temporal distances of the genomes, and to verify a temporal phylogenetic signal in the positive relationship between the recombination-corrected ML tree root-to-tip distances and year of isolation of genomes. To estimate rates of evolution and dates of selected most recent common ancestors (MRCAs; tree nodes), the BEAST v1.7.5 program (Drummond et al., 2012) was used. We applied an HKY+G substitution model with empirical base frequencies, a Bayesian Skyline demographic model with default parameters, and both strict molecular clock and uncorrelated lognormal-relaxed molecular clock models using flat priors between 10^−3^ and 10^−9^ substitutions/site/year as informed by other *S. aureus* studies (Smyth et al., 2010). The basal strains from Argentina were constrained as outgroups, by forcing all other strains to be a monophyletic ingroup. Due to limitations in computational resources, the BEAST runs used three subsamples, each of 101 genomes. Each subsample consisted of 44 genomes chosen to represent early isolates and major nodes on the ML tree, plus 57 randomly selected genomes. Each subsample was run three times, and each run was 100,000,000 steps with sampling every 10,000 steps. Convergence and mixing of the MCMC chains, and the effective sample sizes of parameters (>2374 for the clock.rate parameter for every run of the selected strict clock model), was checked using Tracer v1.6. The first 10% of samples were removed as burn-in, the remaining samples were combined, and maximum clade credibility (MCC) trees were generated using LogCombiner v1.7.5 and TreeAnnotator v1.7.5. Since rate heterogeneity among branches was not extreme in any run of the relaxed clock model (i.e., the 95% credibility interval for the ucld.sd and coeeffvar parameters did not include 1.0), the strict clock model was selected.

### Ancestral State Reconstruction

The evolution of selected traits was studied using ancestral state reconstruction under ML models. The ML analysis was done using the ace function of the ape R package (Paradis et al., 2004). Discrete, two-state characters were investigated including: geography (Central and North America vs. South America), predicted antibiotic resistance (resistant vs. susceptible), and the presence vs. absence of virulence factors, phages, ICEs, and predicted high-impact variants (i.e., frameshifts, start or stop codons gained or lost). Only those traits present in 5% (*n* = 30) to 95% (*n* = 568) of the genomes were investigated. The recombination-corrected ML tree was used as the estimate of the phylogeny. For each trait, both equal rates and all-rates-different models were fit to the data, and models were compared with a likelihood ratio test with one degree of freedom. In no case was the simpler, equal rates model rejected in favor of the more complicated, all-rates-different model.

### Statistical Analysis and Tree Presentation

The basic stats package of R v3.4.2 (R Development Core Team, 2014) was used for two-sided tests of equal means (*t*-tests) and of no correlation between variables (Pearson’s coefficient). Tajima’s *D*, which examines the variance-normalized difference between two estimators of genetic diversity that are sensitive to different population dynamics, was calculated with the pegas (Paradis, 2010) R package. Tests of *D* = 0 were done assuming a beta distribution of the test statistic. Trees were visualized with iTOL (Letunic and Bork, 2016), and the ggtree (Yu et al., 2017) and phytools (Revell, 2012) R packages.

## Results and Discussion

### Outline of the CC5 Phylogeny

**FIGURE 1:**
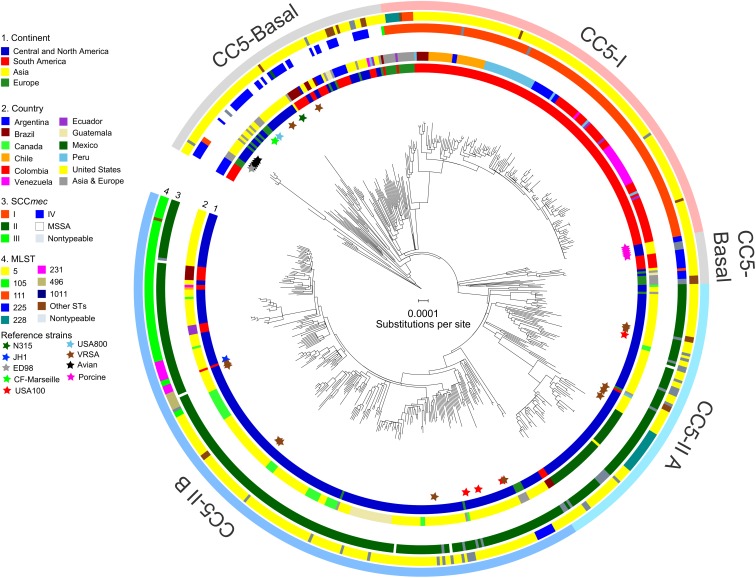
Phylogeny of CC5. The ML phylogeny has recombination-corrected branch lengths and is outgroup-rooted. The major clades of CC5-Basal, CC5-I, and CC5-II are indicated with shading. The statistical support for these clades is described in the text. The positions of reference strains are indicated with stars. The four rings in the direction of inner-outer, show continent (ring 1), country (ring 2), SCC*mec* type (ring 3), and multilocus ST (ring 4) for each genome. STs with less than three genomes were combined into “Other STs” for display purposes. Scale bar indicates number of substitutions per site.

The alignment of 598 genomes of CC5 consisted of 1,081,440 bp present in all genomes, which included 11,961 biallelic SNPs. ML phylogenetic analysis with these sites, followed by correction of tree branch lengths for recombination and outgroup-rooting, resulted in the phylogeny presented in **Figure 1**. Several clades were defined by consideration of their ST-SCC*mec* types, geographic distribution, and statistical support. These clades are described below.

#### CC5-Basal

CC5-Basal was defined as a large paraphyletic clade with a mixture of early-branching MSSA and MRSA predominantly of ST5-IV (SCC*mec* subtypes IVa, IVc, IVg, IVi) (**Figure 1**, light gray shading). This clade was well-dispersed across the Western and Eastern Hemispheres (**Figure 1**, ring 1). The most basal strain in our sample was an MSSA from Argentina, and the next most basal strains were a subclade of ST5-IVa (and one variant of ST5) from Argentina with the *pvl* and *sea* toxin genes that are characteristic of a recently emerged community-associated clone (Sola et al., 2008). Despite the exclusively South American source of these basal strains, ML ancestral state reconstruction did not reliably identify the continent of origin of CC5 (**Supplementary Figure S1**). The Bayesian phylogenetic analysis constrained these basal strains as an outgroup, resulting in maximal posterior probability (PP = 1) for the node, but the ML phylogenetic analysis was unconstrained and still strongly supported the node (SH-like support = 0.984). The Bayesian analysis estimated the MRCA of this sample of CC5 as the late 1930s (**Table 1**).

**Table 1:** Rates of evolution and dates of CC5 clades.

Clade or subclade	Date of MRCA (95% credibility interval)^*a*^	Ratio of recombination to mutation events, *ρ*/*𝜃* (95% credibility interval)^*b*^
All CC5	1938 (1930, 1945) 1937 (1928, 1944) 1938 (1930, 1945)	0.0046 (0.0035, 0.0059)
CC5-Basal	Same as all CC5	0.0016 (0.00063, 0.0029)
CC5-I	1973 (1969, 1977) 1973 (1968, 1977) 1973 (1969, 1977)	0.0046 (0.0019, 0.0086)
…CC5-I expansion	1994 (1992, 1996) 1995 (1993, 1997) 1994 (1992, 1996)	0.0050 (0.0018, 0.0096)
CC5-II	1962 (1957, 1966) 1961 (1955, 1966) 1962 (1956, 1966)	0.0069 (0.0050, 0.0090)
…CC5-II A	Same as CC5-II	0.014 (0.0095, 0.019)
……Mexican subclade	1997 (1994, 2000) 1997 (1994, 1999) 1997 (1994, 2000)	0.019 (0.0089, 0.032)
…CC5-II B	1975 (1970, 1979) 1975 (1970, 1979) 1975 (1970, 1979)	0.0031 (0.0017, 0.0050)
……CC5-II B expansion	1982 (1978, 1986) 1982 (1978, 1986) 1982 (1979, 1986)	0.0029 (0.0014, 0.0049)

*^a^For each clade or subclade, the three results for Date of Most Recent Common Ancestor (MRCA) refer to the three subsamples (see the section “Materials and Methods” for subsampling procedures). The three subsamples had estimated mutation rates (95% credibility intervals) in units of substitutions/site/year of 1.55 × 10^−6^ (1.39 × 10^−6^, 1.71 × 10^−6^), 1.52 × 10^−6^ (1.36 × 10^−6^, 1.68 × 10^−6^), 1.55 × 10^−6^ (1.39 × 10^−6^, 1.70 × 10^−6^), respectively. ^*b*^The ratio of recombination to mutation events was calculated from the complete clade or subclade, without subsampling, using CFML.*

CC5-Basal included the ST5-IVc USA800 clone from the United States (McDougal et al., 2003), and the ST5-I Geraldine clone from France (Dauwalder et al., 2008). Also included are VRSA strains HOU1444-VR from Brazil and VRS3a from the United States, which represented independent acquisitions of vancomycin resistance and confirmed previous analysis (Panesso et al., 2015). CC5-Basal further included ST5-IVa reference strain CF-Marseille from France (Rolain et al., 2009), ST5-II reference strain N315 from Japan (Kuroda et al., 2001), and rare strains of ST5-I and ST5-III from Europe. The Bayesian and ML analyses provided maximal support (PP = 1, SH-like support = 1) for a previously described avian subclade, which included reference strain ED98 (Lowder et al., 2009), and for a separate subclade from porcine sources (Hau et al., 2017). The ML analysis presented a CC5-Basal subclade that included the porcine subclade (**Figure 1**, light gray shading), as a sister to the CC5-II clade described below. However, neither the Bayesian nor ML analyses provided statistical support for this arrangement, so this subclade should be considered to be part of the broader CC5-Basal clade.

#### CC5-I

CC5-I was defined as a large monophyletic clade with SCC*mec* I (**Figure 1**, light red shading). This clade represented one of three separate acquisitions of SCC*mec* I within CC5—the others occurred within the CC5-Basal clade. Most early-branching strains of CC5-I were from Europe, the rest were from South America. The ST228-I South German clone (Ghebremedhin et al., 2005) and related STs ST111 and ST1481 (Budimir et al., 2010) represented these early-branching European strains of CC5-I. The likelihood of a South American origin among CC5-I strains in the Western Hemisphere was >98% (**Supplementary Figure S1**). Bayesian and ML analyses provided maximal support for CC5-I, and its MRCA was estimated as the early 1970s (**Table 1**).

The terminal subclades of CC5-I represented an expansion of the ST5-I Chilean/Cordobes clone (Sola et al., 2002) throughout South America. The expansion node had maximal support in Bayesian and ML analyses, and its MRCA was estimated as the mid-1990s (**Table 1**). The initial branching events of the expansion were unresolved, which was consistent with a rapid expansion where the rate of geographic spread had outpaced the rate of mutation. Moreover, Tajima’s *D* for strains of the expansion was −2.75 (*P* = 3.46 × 10^−5^), which was consistent with a recent expansion because it indicated an excess of rare alleles that would be purged from an older equilibrium population. Subsequent subclades were strongly structured geographically by country within South America, and included strains from Brazil, Chile and nested Argentina strains, Peru, Argentina, Colombia, and nested Venezuela strains (**Figure 1**, ring 2).

#### CC5-II

CC5-II was defined as a very large monophyletic clade with SCC*mec* II (**Figure 1**, light blue shading). This clade represented one of two separate acquisitions of SCC*mec* II within CC5—the other occurred within the CC5-Basal clade on the branch leading to reference strain N315. CC5-II was subdivided into a paraphyletic early-branching subclade, CC5-II A, and a monophyletic terminal subclade, CC5-II B. In the Western Hemisphere, CC5-II strains were mostly from Central and North America, but some strains were from Brazil, Ecuador, and Venezuela. The likelihood of a Central or North American origin among CC5-II strains in the Western Hemisphere was >99% (**Supplementary Figure S1**). This clade had maximal support in Bayesian and ML analyses, and subclade CC5-II B had near maximal support (PP = 1, SH-like support = 0.997). The MRCAs of CC5-II and CC5-II B, respectively, were estimated as the early 1960s and mid-1970s (**Table 1**).

Of the four sampled reference strains of the ST5-II USA100 clone from the United States, one was within CC5-II A and three were within CC5-II B (**Figure 1**), which indicated that this clone was polyphyletic. Furthermore, the separation of ST5-II strains from New York in the United States and from Japan into distinct clades (CC5-II B and CC5-Basal, respectively), indicated that the New York/Japan clone was polyphyletic; in other words, there are separate New York and Japan clones, and not a single New York/Japan clone. Our study focused sampling on the Western Hemisphere, but it will be interesting to see if future studies place Asian SCC*mec* II-positive CC5 strains with strain N315 in CC5-Basal or with the diverse strains in CC5-II. Lastly, of the 11 sampled ST5-II VRSA strains from the United States, five were within CC5-II A and six were within CC5-II B (**Figure 1**, brown stars). Our analysis supported a previous analysis that indicated independent acquisitions of vancomycin resistance for most strains (Kos et al., 2012), but our analysis also highlighted the close relationships of strain pairs VRS1/VRS6, VRS5/VRS7, VRS9/VRS10, and VRS11a/VRS11b. Importantly, no evidence of further dissemination of these VRSA strains was obtained, as ML models of ancestral state reconstruction did not identify strains other than the known VRSAs to descend from vancomycin-resistant MRCAs (nodes). In the absence of vancomycin, the slight fitness burden reported for carriage of the *vanA* operon (Foucault et al., 2009) may be sufficient to impede the dissemination of these strains.

A major feature of CC5-II A was a divergent subclade that included all of the sampled strains from Mexico (**Figure 1**). Early-branching strains of this subclade were from the United States. This subclade had near maximal support (PP = 1, SH-like support = 0.999) in Bayesian and ML analyses, and its MRCA was estimated as the late 1990s (**Table 1**). This time period coincided with the replacement of CC30-MRSA in Mexico by CC5-MRSA, as previously described (Aires De Sousa et al., 2001; Velazquez-Meza et al., 2004; Echaniz-Aviles et al., 2006). This subclade was further subdivided into ST5-II and ST1011-II subclades that showed geographic structure by region within Mexico: ST5-II was from Central and Western Mexico, and ST1011-II was from Central and Northern Mexico.

The ST225-II Rhine-Hesse clone from Central Europe (Schulte et al., 2013) was an early offshoot of CC5-II B. The ST5-II reference strain JH1 from the United States (Mwangi et al., 2007) also nested within CC5-II B. The terminal subclades of CC5-II B represented an expansion of ST5-II, and subsequent proliferation of unnamed clones such as ST105-II, ST125-II, ST231-II, and ST496-II (**Figure 1**, ring 3), in Central and North America. The expansion node had maximal support in Bayesian and ML analyses, and dated to the early 1980s (**Table 1**). Several of the initial branching events of the expansion were unresolved, which was consistent with a rapid expansion. Also, Tajima’s *D* was −2.81 (*P* = 5.91 × 10^−6^), which was consistent with a recent expansion. CC5-II B showed some geographic structure at the country level, such as a subclade with 18 of 21 strains from Guatemala, two subclades with 10–11 strains each from Canada, and a subclade with 18 strains from California in the United States (**Figure 1**, ring 2).

### Relatively Low Recombination Rates in CC5

Our estimate of the ratio of recombination to mutation events in this sample of CC5 (0.0046) (**Table 1**) was several orders of magnitude lower than previously estimated for CC5 (1.08) (Murray et al., 2017). However, the previous estimate detected a large proportion of recombination events in poultry strains and our sample included only six poultry strains for which no recombination events were detected. Sampling differences and the use of different recombination detection methods might explain the discrepancy between our recombination estimate and that of Murray et al. (2017). Our recombination estimate for CC5 was also lower than previously reported for the non-CC5 clones of ST239-III (Castillo-Ramirez et al., 2012) and ST8-IV USA300 (Challagundla et al., 2018); ranges of 0.05–0.29 and 0.12–0.29, respectively. The relatively low recombination rate estimated here using CFML was confirmed with an independent analysis using Gubbins (Croucher et al., 2015): 58 recombination events were detected by CFML and 29 were detected by Gubbins. In our sample, CC5-II A had a significantly higher ratio of recombination to mutation events compared to other CC5 clades (**Table 1**). This result was driven by a relatively large number of recombination events in the Mexican subclade. Thus, our analysis did not support the hypothesis that CC5 was generally more promiscuous than other *S. aureus* genetic backgrounds. Other explanations for why VRSA strains have repeatedly appeared in CC5 should be sought, such as whether CC5 has a unique gene regulatory environment that favors *vanA* expression or a unique ecological niche that favors interactions with *Enterococcus* spp.

### Evidence for an Antibiotic Resistance-Toxicity Tradeoff in CC5

The full range of detected mutations and genes associated with antibiotic resistance, and selected virulence factors and mobile genetic elements, is provided in **Supplementary Table S1**. Our discussion is focused on those traits that are variably distributed among CC5 clades in the Western Hemisphere. Besides the high prevalence of resistance to β-lactams in all clades, CC5-I and CC5-II had a high prevalence of resistance to fluoroquinolones, macrolides, and lincosamides, and CC5-I also had a high prevalence of resistance to aminoglycosides (**Table 2**). The most common resistance mechanism to fluoroquinolones was the “double-serine mutations,” Ser84Leu in *gyrA* and Ser80Phe in *grlA*, that have been noted to occur in other successful MRSA clones (Fuzi et al., 2017). These double-serine mutations accounted for fluoroquinolone resistance in 141 of 147 (96%) resistant strains of CC5-I, and 107 of 239 (45%) resistant strains of CC5-II. Of note, CC5-II B had a lower prevalence of fluoroquinolone resistance compared to CC5-II A (58% vs. 98%, respectively) (**Table 2**), and the main mechanism of resistance in CC5-II B was the single mutation in *gyrA* that accounted for 117 of 141 (83%) resistant strains of CC5-II B. The most common resistance mechanism to macrolides and lincosamides was the *ermA* gene: alone accounting for 139 of 144 (97%) resistant strains of CC5-I, and 268 of 342 (78%) resistant strains of CC5-II. The *ermA* gene is known to be carried on Tn*554* on the SCC*mec* II element (Kuroda et al., 2001), and it occurred on Tn*554* elsewhere in the chromosome of the examined strains with SCC*mec* I elements. Aminoglycoside resistance, which occurred mostly in CC5-I, was exclusively attributed to the *aacA*–*aphD* bifunctional gene in our sample, and this gene occurred on composite transposon Tn*4001* in examined strains. Mobile genetic elements also carried some rare antibiotic resistance genes. For example, ICE element (also known as conjugative transposon) Tn*916* accounted for all eight strains with *tetM*-mediated tetracycline resistance. The other family of ICE elements known in *S. aureus*, ICE*6013*, was not observed to carry any antibiotic resistance genes and was common among the avian subclade of CC5-Basal (**Table 2**).

**Table 2:** Prevalence of selected traits in CC5 clades.

	Trait	Clade or subclade^*a*^			
		CC5-Basal (*n* = 107)	CC5-I (*n* = 148)	CC5-II A (*n* = 100)	CC5-II B (*n* = 243)
Antibiotic resistances	CIP	33 (30.84)	147 (99.32)	98 (98)	141 (58.02)
	ERY	28 (26.17)	144 (97.3)	99 (99)	243 (100)
	CLIN	15 (14.02)	144 (97.3)	99 (99)	243 (100)
	FUS	1 (0.93)	1 (0.68)	0 (0)	3 (1.23)
	GENT	16 (14.95)	143 (96.62)	19 (19)	13 (5.35)
	OXA	63 (58.88)	148 (100)	99 (99)	239 (98.35)
	MUP	1 (0.93)	1 (0.68)	6 (6)	10 (4.12)
	PEN	89 (83.18)	148 (100)	100 (100)	241 (99.18)
	RIF	8 (7.48)	11 (7.43)	7 (7)	10 (4.12)
	TET	20 (18.69)	1 (0.68)	1 (1)	4 (1.65)
	TMP	10 (9.35)	1 (0.68)	16 (16)	12 (4.94)
	VANC	2 (1.87)	0 (0)	5 (5)	6 (2.47)
Virulence factors	*etb*	9 (8.41)	0 (0)	0 (0)	0 (0)
	*lukDE*	104 (97.2)	145 (97.97)	89 (89)	229 (94.24)
	*lukFS*	6 (5.61)	0 (0)	0 (0)	0 (0)
	*sea*	45 (42.06)	16 (10.81)	38 (38)	3 (1.23)
	*seb*	7 (6.54)	0 (0)	0 (0)	6 (2.47)
	*sec*	4 (3.74)	0 (0)	0 (0)	0 (0)
	*sed*	20 (18.69)	0 (0)	24 (24)	163 (67.08)
	*seg*	106 (99.07)	142 (95.95)	90 (90)	226 (93)
	*sei*	106 (99.07)	144 (97.3)	87 (87)	226 (93)
	*sej*	23 (21.5)	0 (0)	23 (23)	163 (67.08)
	*sek*	2 (1.87)	1 (0.68)	0 (0)	1 (0.41)
	*sel*	4 (3.74)	0 (0)	0 (0)	0 (0)
	*sem*	107 (100)	144 (97.3)	91 (91)	231 (95.06)
	*sen*	106 (99.07)	139 (93.92)	89 (89)	225 (92.59)
	*seo*	107 (100)	146 (98.65)	91 (91)	231 (95.06)
	*sep*	58 (54.21)	7 (4.73)	63 (63)	12 (4.94)
	*seq*	2 (1.87)	0 (0)	0 (0)	1 (0.41)
	*ser*	22 (20.56)	1 (0.68)	23 (23)	163 (67.08)
	*sev*	107 (100)	144 (97.3)	87 (87)	228 (93.83)
	*tst1*	12 (11.21)	1 (0.68)	1 (1)	1 (0.41)
	ψ*ent2*	106 (99.07)	143 (96.62)	90 (90)	228 (93.83)
	*fnbB*	105 (98.13)	5 (3.38)	97 (97)	238 (97.94)
	*sdrE*	99 (92.52)	143 (96.62)	89 (89)	229 (94.24)
	*chp*	70 (65.42)	118 (79.73)	85 (85)	223 (91.77)
	*scn*	85 (79.44)	133 (89.86)	91 (91)	226 (93)
	*sak*	86 (80.37)	133 (89.86)	92 (92)	230 (94.65)
Phages	ϕSa12*int*	1 (0.93)	0 (0)	0 (0)	2 (0.82)
	ϕSa1*int*	39 (36.45)	3 (2.03)	41 (41)	194 (79.84)
	ϕSa2*int*	31 (28.97)	110 (74.32)	61 (61)	132 (54.32)
	ϕSa3*int*	92 (85.98)	134 (90.54)	90 (90)	231 (95.06)
	ϕSa4*int*	10 (9.35)	2 (1.35)	16 (16)	13 (5.35)
	ϕSa5*int*	6 (5.61)	2 (1.35)	6 (6)	66 (27.16)
	ϕSa6*int*	14 (13.08)	6 (4.05)	40 (40)	20 (8.23)
	ϕSa7*int*	18 (16.82)	147 (99.32)	65 (65)	75 (30.86)
	ϕSa9*int*	0 (0)	0 (0)	1 (1)	0 (0)
	ϕSPβ*int*	0 (0)	0 (0)	0 (0)	3 (1.23)
ICEs	ICE*6013*_sf1	23 (21.49)	1 (0.68)	6 (6)	6 (2.47)
	ICE*6013*_sf5	8 (7.48)	0 (0)	0 (0)	0 (0)
	ICE*6013*_sf6	6 (5.61)	0 (0)	0 (0)	0 (0)
	Tn*916*	6 (5.61)	0 (0)	0 (0)	2 (0.82)

*^a^Numbers in cells refer to the number (percentage) of genomes in the indicated clade or subclade that have the indicated trait.*

Common virulence factors of CC5 included the *sak*, *scn*, and *chp* genes of the immune evasion gene cluster (IEC) present on phage ϕSa3, and *seg*, *sei*, *sem*, *sen*, *seo*, and ψ*ent* of the enterotoxin gene cluster (EGC) as well as *lukDE* present on genomic island νSaβ (**Table 2**). CC5-Basal appeared to have a more diverse array of toxins, and the *pvl* (*lukFS*), *sec*, *sel*, and *etb* toxins occurred solely in this clade. CC5-I was unique in having a low prevalence of the *fnbB* adhesin gene; it was present in only 3% of CC5-I strains, and in >96% of strains of other clades (**Table 2**). On the other hand, the plasmid-borne *sed*, *sej*, *ser* toxins were most common in CC5-II B strains. Phages also showed some differences in CC5 clade distribution: phages ϕSa2 and ϕSa7 were most common in CC5-I strains, and phage ϕSa1 was most common in CC5-II B strains (**Table 2**).

We tested the hypothesis that antibiotic resistances and toxins were randomly distributed throughout CC5 and across time by comparing the average number of antibiotic resistances and toxins per genome in the various clades and by examining these traits with distance from the root of the CC5 tree. CC5-Basal had significantly fewer antibiotic resistances and more toxins than the other clades (**Table 3**). CC5-I had the opposite pattern, significantly more antibiotic resistances and fewer toxins than the other clades. CC5-II also had significantly more antibiotic resistances, but it had more toxins due to greater toxin acquisition in CC5-II B (e.g., *sed*, *sej*, *ser*) that offset the loss in CC5-II A (**Table 3**). Overall, the number of antibiotic resistances per genome increased with distance from root, and the number of toxins per genome decreased with distance from root (**Table 3**). A similar trend of increased number of antibiotic resistances with distance from root has been reported previously for CC8 (Strommenger et al., 2014). While these results highlight the importance of antibiotic resistance to the evolution of CC5, they are subject to a statistical caveat. Not all of the data points are independent because mobile genetic elements can carry multiple linked antibiotic resistance or toxin genes and affect multiple traits in a single evolutionary event; for example, *ermA* mediates both macrolide and lincosamide resistance, and the EGC encodes multiple enterotoxins.

**Table 3:** Comparisons of antibiotic resistance and toxin traits in CC5 clades.

Clade or subclade	Number of genomes	Average number per genome (SD)^*a*^		Correlation between number per genome and root-to-tip distance^*b*^	
		Antibiotic resistances^*c*^	Toxins^*d*^	Antibiotic resistances^*c*^	Toxins^*d*^
All CC5	598	4.8 (1.6)	9.2 (2.1)	0.394^∗∗∗∗^	−0.326^∗∗∗∗^
CC5-Basal	107	2.7 (2.0)^∗∗∗∗^	9.9 (2.1)^∗∗∗∗^	0.029	−0.295^∗∗^
CC5-I	148	6.0 (0.6)^∗∗∗∗^	7.9 (1.0)^∗∗∗∗^	−0.178^∗^	0.126
CC5-II	343	5.0 (0.9)^∗∗^	9.4 (2.3)^∗∗∗^	−0.017	−0.194^∗∗∗^
…CC5-II A	100	5.5 (1.0)^∗∗∗∗^	8.9 (2.4)	−0.224^∗^	−0.037
…CC5-II B	243	4.8 (0.8)	9.7 (2.2)^∗∗∗∗^	−0.089	−0.284^∗∗∗∗^

*^a^Standard deviation (SD), test is for equal means of clade or subclade vs. all others. ^b^Test is for zero correlation, root-to-tip distance is from CFML tree. ^c^From a possible total of 12 predicted resistances (CIP, CLIN, ERY, FUS, GENT, OXA, MUP, PEN, RIF, TET, TMP, VANC). ^d^From a possible total of 20 toxins (etb, lukDE, lukFS, sea, seb, sec, sed, seg, sei, sej, sek, sel, sem, sen, seo, sep, seq, ser, sev, tst1). ^∗^P < 0.05, ^∗∗^P < 0.01, ^∗∗∗^P < 0.001, ^∗∗∗∗^P < 0.0001.*

### Convergent Genomic Changes Associated With Expansions of CC5

**FIGURE 2:**
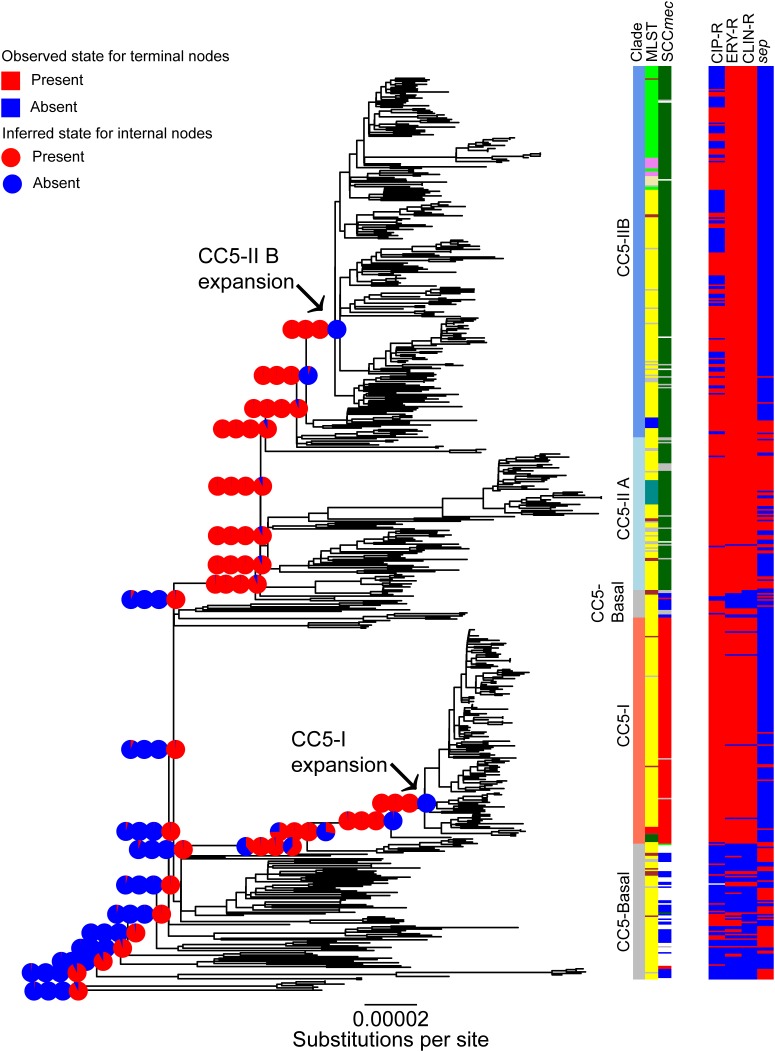
Convergent evolution within CC5. The ML phylogeny of **Figure 1** is shown. The major clades, multilocus ST, and SCC*mec* type are shown for reference purposes and colored as in **Figure 1**. The CC5-I and CC5-II B expansion nodes are indicated with arrows. ML ancestral state reconstruction is shown along the phylogenetic backbone leading to the two expansion nodes; the four pie-charts at each of the backbone nodes indicate the likelihood of (in left-to-right order), fluoroquinolone, macrolide, and lincosamide resistance (red) or susceptibility (blue), and the presence (red) or absence (blue) of the *sep* toxin gene. Table adjacent to the tree indicates these traits in each genome. Scale bar indicates number of substitutions per site.

The evolution of selected traits was studied in more detail under an ML model of ancestral state reconstruction. A major finding of this analysis was that resistance to fluoroquinolones, macrolides, and lincosamides was gained, and the *sep* toxin gene was lost, independently, along the phylogenetic backbone leading to the expansion nodes of CC5-I and CC5-II B (**Figure 2**, pie-charts). The likelihood of the nodes that precede CC5-I and CC5-II B having these resistances was <5–6% for fluoroquinolones and <1% for macrolides and lincosamides, and >98% for the presence of *sep*. By the time of the expansion nodes of CC5-I and CC5-II B, the likelihood of having all three resistances was >99%, and <1% for the presence of *sep* (**Figure 2**, pie-charts).

The independent evolution of the same traits from different starting points on the CC5 phylogeny (i.e., convergence), and the close timing of their evolution with independent expansions, suggests a causal relationship. Use of β-lactams, macrolides, and fluoroquinolones rank highly among antibiotics in the United States, Canada, and Brazil (Center for Disease Dynamics et al., 2015), and likely other countries in South America. Thus, the gain of these resistances may reflect a common selective pressure in the Western Hemisphere. As can be seen in **Figure 2** (bar charts), some of the strains that appeared after the expansion of CC5-II B have subsequently lost fluoroquinolone resistance. As indicated above, many of the CC5-II B strains that retained fluoroquinolone resistance have retained the *gyrA* resistance allele but have lost the *grlA* resistance allele. Fluoroquinolone resistance mutations and especially the *grlA* mutation, have been shown to negatively impact the fitness of *S. aureus* strains in the absence of antibiotic (Horváth et al., 2012; Knight et al., 2012). However, these fitness effects may be mitigated in the presence of sub-inhibitory levels of the antibiotic (Gustave et al., 2018). These observations suggest that fluoroquinolone resistance may have an important role in the initial phase of epidemic spread CC5-MRSA clones, but less of a role in the subsequent phase of endemic residence in hospitals. Alternatively, there may have been efforts to reduce fluoroquinolone use in recent years, which in turn may have alleviated the selective pressure for clones to remain resistant. The CC5-II B expansion began approximately one decade earlier than the CC5-I expansion (**Table 1**), so it will be interesting to see if CC5-I begins to lose fluoroquinolone resistance and the *grlA* resistance allele over time as has happened in CC5-II B.

The association of the CC5-I and CC5-II B expansions with independent losses of the *sep* toxin gene was unexpected. This gene is present on the IEC of phage ϕSa3. Its loss preceding the CC5-I and CC5-II B expansions does not represent loss of the phage, since the majority of strains retained the phage and other virulence factors of the IEC such as *sak*, *scn*, and *chp* (**Table 2**). The completely sequenced reference strains N315 and JH1, respectively, provide full sequences of the IEC before and after loss of *sep*. A 1.75 kb region of the IEC that includes the *sep* gene was noted to be variably present in *S. aureus* by van Wamel et al. (2006). To our knowledge, the mechanism of *sep* excision from the IEC is unknown, and the function of *sep* is unstudied beyond its characterization as a superantigen with emetic activity (Omoe et al., 2005, 2013). In one study, the presence of *sep* was identified as the only *S. aureus* virulence factor among 30 tested, to associate with bacteremia in hospitalized MRSA carriers (Calderwood et al., 2014). While *sep* and other genome variations might influence the risk of bacteremia, those types of invasive infections are dead-ends for MRSA transmission.

The overall trend of decreased number of toxins per genome with distance from root, and parallel losses of *sep* in particular, suggests that the CC5 expansions occurred with strains that were less virulent than their precursors. Additional observations that support this notion come from an analysis of 35 high-frequency, high-impact mutations (listed in **Supplementary Table S1**). A total of 14 of these mutations occurred in CC5-I by the time of the expansion and three more occurred after the expansion. One of these was a frameshift mutation in the *sasG* adhesin gene, which also occurred in CC5-I strains that lacked the *fnbB* gene. Loss of *sasG* and *fnbB* function would be expected to result in reduced virulence especially in biofilm-associated infections (Vergara-Irigaray et al., 2009; Geoghegan et al., 2010). In CC5-II B, only two high-frequency, high-impact mutations occurred by the time of the expansion, but four occurred afterward. One of these mutations resulted in the loss of the stop codon of the *srtA* sortase gene, which is a virulence factor that attaches surface proteins with the LPXTG motif to *S. aureus*’ peptidoglycan cell wall. Loss of sortases function would be expected to result in reduced virulence (Mazmanian et al., 2000). One interesting gain-of-function change in CC5-II B was a frameshift mutation that restored the start codon of the toxin component of the *axe1*/*txe1* toxin/antitoxin system. Expression of this system in CC8 strain Newman is increased after exposure to subinhibitory concentrations of erythromycin and tetracycline (Donegan and Cheung, 2009), but it has not been studied in CC5 to our knowledge.

## Concluding Remarks

CC5-MRSA in the Western Hemisphere are highly diverse, even those strains that share the same ST-SCC*mec* type and circulate in the same country. More precise definitions for commonly sampled clones such as the USA100 and New York/Japan clones, and likely other clones such as USA800, are needed because different strains with those labels may have shared a MRCA > 50 years ago and may have divergent genomes. Our study provides the first systematic effort at organizing this diversity from a phylogenomic perspective and it provides a robust landmark for future genome studies of CC5-MRSA strains. The geographic structure of CC5 that is evident at the continent, country, and even region levels in some cases, suggests that a more precise delineation of the patterns of geographic spread of CC5-MRSA clones may be possible. MRSA clones of clinical relevance branch at nearly all time depths in the CC5 phylogeny; from the earliest-branching ST5-IV clones within the CC5-Basal clade that are an emerging problem in hospitals and communities in South America, to the latest-branching ST5-II clones within the CC5-II B clade that are a continuing problem in hospitals in Central and North America. Our analysis shows relatively low rates of recombination in CC5, which indicates that the propensity of CC5 to acquire *vanA*-mediated vancomycin resistance is not likely due to enhanced promiscuity and prompts study of other potential mechanisms. In tracing the evolution of selected traits of CC5, we discovered that some of the genomic changes that occurred prior to expansions of the CC5-I and CC5-II B clades represented instances of convergent evolution. While the convergent acquisition of resistance to widely prescribed antibiotics is a recurring theme in the history of successful MRSA clones, the convergent loss of the *sep* toxin and the trend of decreasing number of toxins with distance from the CC5 tree root and loss of other virulence factors is a unique finding. Taken together, our results suggest that more antibiotic-resistant and less virulent CC5-MRSA clones may be better able to spread geographically.

## Data Availability

Sequence reads are available from NCBI Bioproject PRJNA224189, PRJNA454482, and PRJNA291213. Accession numbers for sequences are provided in **Supplementary Table S1**.

## Data Deposition

NCBI Bioproject (http://www.ncbi.nlm.nih.gov/bioproject) PRJNA224189, PRJNA454482, and PRJNA291213.

## Author Contributions

LC, MF, and DR conceived and designed the study. JR, DS, GE-A, MV-M, NF, CA, LD, and DR contributed bacterial strains or DNA. JR, MF, SC, LC, SR, BH, LD, and DR performed the genome sequencing. LC, JR, IR, MF, SC-R, MC, BH, PP, LD, and DR performed the analysis. LC and DR drafted the manuscript. All authors critically reviewed and approved the manuscript.

## Conflict of Interest Statement

The authors declare that the research was conducted in the absence of any commercial or financial relationships that could be construed as a potential conflict of interest.

## Footnotes

1 http://www.broadinstitute.org/software/allpaths-lg/blog

2 http://www.broadinstitute.org/software/pilon/

3 https://pubmlst.org/saureus/

4 https://github.com/tseemann/mlst

5 http://www.sccmec.org

## Supplementary Material

FIGURE S1 Geographic structure within CC5.

TABLE S1 Characteristics of CC5 genome sequences.

TEXT S1 Genome sequencing procedures for the Broad Institute.
