# Supplementary material for: Phylogenomic Classification and the Evolution of Clonal Complex 5 Methicillin-Resistant Staphylococcus aureus in the Western Hemisphere
Source: Front Microbiol. 2018 Aug 22;9:1901. doi: 10.3389/fmicb.2018.01901 (PMC6113392; doi:10.3389/fmicb.2018.01901)
Supplement: TEXT S1 — Genome sequencing procedures for the Broad Institute. [file Data_Sheet_1.docx]

**Supplementary Text 1. Genome sequencing procedures for the Broad Institute.**

DNA Samples were received and checked into the Broad Institute of MIT and Harvard’s Sample repository. Samples were measured using PicoGreen^®^ dsDNA quantitation reagent, the ultra-sensitive fluorescent nucleic acid stain for quantitating double-stranded DNA. Once volumes, concentrations and a DNA yield of >0.25ug were obtained, samples were moved into library construction.

Illumina fragment libraries were generated as previously described (Bentley et al. 2008) with the following modifications. For each sample, 100 ng of genomic DNA was sheared to 200 bp in size using a Covaris LE220 instrument (Covaris, MA) with the following parameters: temperature: 7–9 °C; duty cycle: 20%; intensity: 5; cycles per burst: 200; time: 90 s; shearing tubes: Crimp Cap microTUBES with AFA fibers Covaris, MA). DNA fragments were end repaired, 3′ adenylated, ligated with indexed Illumina sequencing adapter, and PCR enriched, as previously described (Fisher et al. 2011). The resulting Illumina fragment sequencing libraries were normalized and were size selected to contain inserts of 180 bp ± 3% in length using a Pippen Prep system (Sage Science, MA) following the manufacturer’s recommendations.

Illumina sequencing libraries were quantified using quantitative PCR (KAPA Biosystems, MA) following the manufacturer’s recommendations. Libraries were normalized to 2 nM and denatured using 0.1 N NaOH. Sequencing Flowcell cluster amplification was performed according to the manufacturer’s recommendations using the V3 TruSeq PE Cluster Kit and V3 TruSeq Flowcells (Illumina, CA). Flowcells were sequenced with 101 base paired end reads on a Illumina HiSeq2000 instrument, using V3 TruSeq Sequencing by synthesis kits and analyzed with the Illumina RTA v1.12 pipeline (Illumina, CA). Libraries were sequenced on the Illumina HiSeq 2000 instrument aiming for 100X coverage each.

References for Supplemental Text 1.

Fisher S., Barry A., Abreu J., Minie B., Nolan J., Delorey T.M., et al. (2011). A scalable, fully automated process for construction of sequence-ready human exome targeted capture libraries. Genome. Biol. 12, R1.

Bentley D.R., Balasubramanian S., Swerdlow H.P., Smith G.P., Milton J., Brown C.G., et al. (2008). Accurate whole human genome sequencing using reversible terminator chemistry. Nature. 456, 53-99.
